# A comparison between invasive and noninvasive measurement of the Hypotension Prediction Index: A post hoc analysis of a prospective cohort study

**DOI:** 10.1097/EJA.0000000000002082

**Published:** 2024-10-16

**Authors:** Santino R. Rellum, Eline Kho, Jimmy Schenk, Björn J.P. van der Ster, Alexander P.J. Vlaar, Denise P. Veelo

**Affiliations:** From the Department of Anaesthesiology (SRR, EK, JS, BJPvdS, DPV), Department of Intensive Care, Amsterdam UMC, University of Amsterdam, Amsterdam Cardiovascular Sciences (SRR, EK, JS, APJV) and Department of Epidemiology and Data Science, Amsterdam UMC, University of Amsterdam, Amsterdam Public Health, Amsterdam, the Netherlands (JS)

## Abstract

**BACKGROUND:**

Clinical trials and validation studies demonstrate promising hypotension prediction capability by the Hypotension Prediction Index (HPI). Most studies that evaluate HPI derive it from invasive blood pressure readings, but a direct comparison with the noninvasive alternative remains undetermined. Such a comparison could provide valuable insights for clinicians in deciding between invasive and noninvasive monitoring strategies.

**OBJECTIVES:**

Evaluating predictive differences between HPI when obtained through noninvasive versus invasive blood pressure monitoring.

**DESIGN:**

Post hoc analysis of a prospective observational study conducted between 2018 and 2020.

**SETTING:**

Single-centre study conducted in an academic hospital in the Netherlands.

**PATIENTS:**

Adult noncardiac surgery patients scheduled for over 2 h long elective procedures. After obtaining informed consent, 91 out of the 105 patients had sufficient data for analysis.

**MAIN OUTCOME MEASURES:**

The primary outcome was the difference in area under the receiver-operating characteristics (ROC) curve (AUC) obtained for HPI predictions between the two datasets. Additionally, difference in time-to-event estimations were calculated.

**RESULTS:**

AUC (95% confidence interval (CI)) results revealed a nonsignificant difference between invasive and noninvasive HPI, with areas of 94.2% (90.5 to 96.8) and 95.3% (90.4 to 98.2), respectively with an estimated difference of 1.1 (−3.9 to 6.1)%; *P* = 0.673. However, noninvasive HPI demonstrated significantly longer time-to-event estimations for higher HPI values.

**CONCLUSION:**

Noninvasive HPI is reliably accessible to clinicians during noncardiac surgery, showing comparable accuracy in HPI probabilities and the potential for additional response time.

**TRIAL REGISTRATION:**

Clinicaltrials.gov (NCT03795831) https://clinicaltrials.gov/study/NCT03795831

**KEY POINTS**

- Invasive and noninvasive HPI have similar discriminatory ability in predicting hypotension from nonhypotension.
- Noninvasive HPI may provide additional intervention time for alerting thresholds above an index of 55.
- Some patients show differences in amount of detected hypotensive events between invasive and noninvasive blood pressures.
- HPI may produce an AUC below 70% in certain patients, but specific patient characteristics explaining this could not be identified.

## Introduction

Haemodynamic instability is a problem that arises in various patients and hospital settings. Timely detection of hypotension through continuous monitoring can facilitate improved management.^1^ Clinical trials implementing the Hypotension Prediction Index (HPI)^2^ demonstrated a substantial reduction in the degree of hypotension when this was added to continuous monitoring. In this regard, noninvasive HPI has proved successful in awake patients,^3^ and it has performed promisingly in validation studies.^4,5^ However, the criteria for choosing invasive or noninvasive HPI and potential performance differences remain unclear.

Several different patient groups, such as those in the emergency department, postanaesthesia care unit, or cardiac care unit, could benefit from HPI. The availability of a noninvasive option removes barriers for implementing a HPI protocol in these cohorts, where invasive monitoring, with cannulation risks, may outweigh the possible benefit of improved haemodynamic stability.^6–8^ It is reassuring that blood pressure readings between invasive and noninvasive datasets are comparable^9^; however, it is not immediately evident that their HPI predictions perform equally well. Therefore, a direct comparison between the performance of their HPI values is necessary to elucidate differences and guide clinicians in determining its use in specific individuals and settings. Recent discussions on HPI validation have highlighted potential shortcomings in receiver-operating characteristics (ROC) analyses, as these supposedly overestimated HPI's predictive ability.^10–12^ However, when evaluating HPI against itself, this concern is eliminated, as any difference between the two will remain.

The primary objective of this retrospective observational study is to determine whether HPI's predictive performance based on continuous invasive waveform data significantly differs from noninvasively obtained HPI using ROC characteristics. Additionally, in case of predictive underperformance in either predictor, we will explore influencing factors.

## Methods and analysis

### Ethics

This was an exploratory analysis of an investigator-initiated, prospective feasibility study, conducted at the Amsterdam UMC, location AMC, the Netherlands (NCT03795831), with approval (N° W17_362) from the ethical committee METC Amsterdam UMC, Amsterdam, the Netherlands on the 28th of September 2017. The research followed the principles of the Declaration of Helsinki and the Good Clinical Practice guidelines. Patients provided written, informed consent and were recruited between April 2018 and October 2020. Findings are reported following the TRIPOD guidelines,^13^ and analysed indices are presented in accordance with the STARD diagram.^14^

### Patients

The original study included adults (≥18 years) scheduled for elective noncardiac surgery requiring an arterial line, and prioritised procedures with a planned duration of over 2 h. Exclusion criteria were conditions anticipated to substantially interfere with the transmission of arterial pressures to the finger, including recent finger fractures, recent finger surgery, severe oedema and severe burns of the skin. Conditions involving moderate vasoconstriction (cold hands) were integral to the feasibility study, and therefore not excluded. This subgroup shows overlap with the group initially incorporated into the development of the HPI algorithm.^2^

### Study procedures and data collection

In the operating room, all patients had a 20-G radial arterial catheter inserted for continuous haemodynamic monitoring using a FloTrac pressure transducer recorded on an EV1000^TM^ monitor (Edwards Lifesciences, Irvine, California, USA). Simultaneously, noninvasive data were obtained through a finger cuff connected to a ClearSight system (Edwards Lifesciences). The haemodynamic waveform datasets were stored in a password-protected sub-directory.

The invasive and noninvasive datasets were synchronised. The invasive and noninvasive datasets were synchronised at the timepoint when both databases were receiving data, and ended at the timepoint where one of the datasets was the first to stop receiving data. If this total selection had a duration of at least 2 h, the dataset was used; otherwise, it was discarded. Other reasons for discarding datasets included incomplete data in either dataset (invasive or noninvasive) because of technical reasons, or if consent was withdrawn.

### Hypotension Prediction Index performance classification

**Fig. 1:**
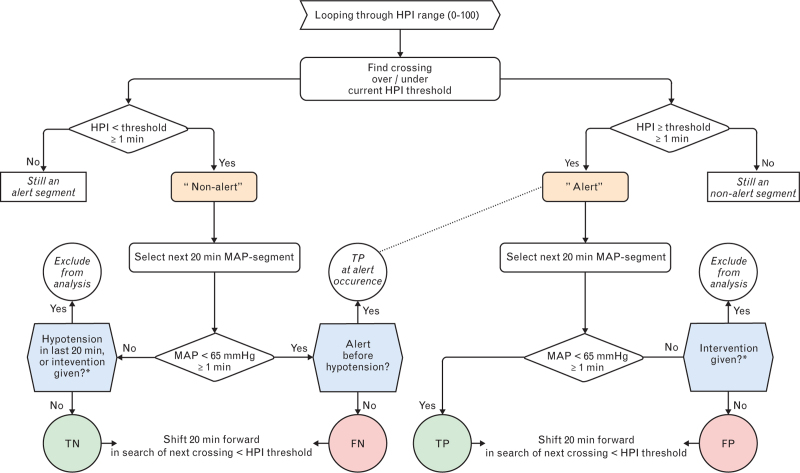
HPI classification flowchart evaluating the following 20 minutes.

FN, false-negative; FP, false-positive; HPI, Hypotension Prediction Index; MAP, mean arterial pressure; min, minute(s); TN, true-negative; TP, true-positive. ^∗^haemodynamic intervention is assumed cases where MAP changes ≥ 5 mm Hg within 20s or ≥ 8 mm Hg within 2 minutes from a baseline MAP < 70 mmHg.

HPI is a logistic regression-based model that generates an index (0 to 100), indicating the probability of forthcoming hypotension. The HPI performance classification followed previous validation studies using a forward selection analysis, assigning predictive classifiers to 20 min timeframes,^4,15^ as any predictions further ahead in time were not deemed clinically relevant. Binary classifiers were assigned for each HPI threshold separately, to obtain the performance for every available alerting threshold. In this regard, an alert was defined as a value above or equal to the evaluated threshold for at least a minute. Hypotension was defined as a mean arterial pressure (MAP) below 65 mmHg for at least 1 min, persisting until the blood pressure stabilised at or above 65 mmHg for a minimum of 1 min. Consequently, an HPI alert followed by hypotension within 20 min was classified as true-positive (Fig. 1). Conversely, an HPI alert not followed by hypotension within 20 min was classified as false-positive. HPI nonalerts combined with normotension in the 20 min prior and without subsequent hypotension were true-negative, whereas, in cases of hypotension, they were false-negative. As with earlier studies,^2,4,15–17^ we excluded the 20 min timeframe when MAP was less than 70 mmHg and suddenly changed (≥ 5 mmHg within 20 s and ≥8 mmHg within 2 min), correcting for possible haemodynamic interventions. After class allocation, the timeframe selection was shifted forward by 20 min. As an exception, if a false-negative class was assigned, but an HPI alert started before hypotension emerged, the false-negative class was discarded. Based on the obtained classes over the full range of HPI, the area under the ROC curve (AUC) was calculated for both datasets.

### Sample size calculation

The sample size for this study was determined by the parent feasibility study, which followed ISO 81060-2 standards requiring a minimum of 30 patients for noninvasive sphygmomanometers. The parent study tripled this amount to capture a comprehensive dataset sufficient for determining the feasibility of noninvasive measures.

### Study outcomes and statistical analyses

The primary outcome was the difference in AUC between the two datasets. This was determined through forward selection analysis using a 20 min timeframe. Exploratory outcomes assessed the proportions on both sides of a 70% cut-off value for AUC (poor and acceptable AUC outcomes) to determine differences in patient characteristics. Additionally, average time-to-event estimations were calculated for each HPI threshold. These estimations represent the time from the onset of an HPI alert to the onset of a hypotensive event. Finally, the patient characteristics were evaluated for those with and without hypotensive events.

To compare different AUCs, the number of classifiers per patient for each dataset was bootstrapped with 2000 iterations. The 95% of data closest to the mean were used to obtain the standard deviation (SD) to perform paired two-sided *t* tests. Depending on the distribution, continuous data are presented as medians with first and third quartile [Q1 to Q3] or as mean ± SD or 95% confidence interval (CI). Normality of distribution was visually assessed based on histograms and Q–Q plots. Differences between paired continuous data were assessed with a *t* test or Wilcoxon signed rank test whenever appropriate. Differences between medians were estimated using the Hodges’–Lehmann method. Categorical data are displayed as frequencies with percentages. The *α*-threshold for statistical significance was ≤ 0.05 for all tests. Analyses were performed using MATLAB (Version 2022b, The Mathworks Inc., Natick, Massachusetts, USA).

## Results

**Fig. 2:**
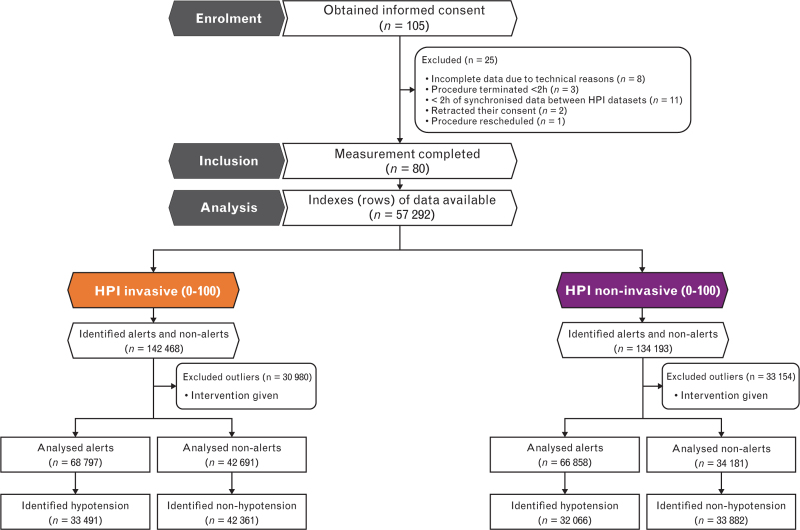
Flowchart number of participants and indexes analysed per dataset.

The original feasibility study excluded 25 patients after informed consent was obtained (Fig. 2). The remaining 80, primarily ASA class II general surgery patients, without missing data were analysed (Table 1). There was an even distribution of open and laparoscopic procedures with a median of 3.5 h of data [3.0 to 4.7], and 40% were female patients. The median doses of drugs administered that could affect haemodynamic status per patient were: sufentanil 80 [50 to 118] μg, propofol 160 [100 to 1825] mg, esketamine 20 [0 to 25] mg, ephedrine 7.8 [0 to 10] mg, phenylephrine 100 [0 to 300] μg and norepinephrine 1077 [574 to 1333] μg. In total, 77 (96.3%) patients received a norepinephrine infusion during the procedure, and 67 (83.8%) required additional vasopressor boluses. None received inotropic agents or required support from a haemodynamic assist device (intra-aortic balloon pump, extracorporeal membrane oxygenation). The median volume of fluids administered was 1518 [1000 to 2355] ml, including solutions such as NaCl 0.9%, Plasmalyte, Sterofundin, Tetraspan and Volulyte. Additionally, three patients required erythrocyte transfusion, whereas one patient received plasma and thrombocytes.

The flowchart shows all indexes analysed for each HPI threshold from 0 to 100. Identified alerts represent every alert evaluated at each threshold, leading to identified hypotension if correct. The identified hypotension does not represent the number of hypotensive events in each dataset. Instead, it is the cumulative count of events recognised by each available alert across the entire HPI range, with each event potentially being recognised by multiple HPI alert thresholds.

**Table 1:** Patient characteristics compiled for area under the receiver-operating characteristics curve values above or equal to 70% or below 70%

	Invasive	Noninvasive			Invasive	Noninvasive		
Category	AUC <70% (*n* = 6)	AUC <70 (*n* = 10)	Difference	*P* value	AUC ≥70 (*n* = 64)	AUC ≥70% (*n* = 59)	Difference	*P* value
Age (years)	61.2 ± 18.5	57.4 ± 16.8	−3.8	0.679	62.3 ± 11.3	62.5 ± 10.8	0.2	0.920
Sex (female)	6 (100)	5 (50)	−50%	0.037	20 (31.3)	24 (40.7)	9.4	0.276
Weight (kg)	65.5 ± 8.5	72.2 ± 13.3	6.7	0.291	78.5 ± 13.7	78.6 ± 14.4	0.1	0.969
Height (cm)	168.8 ± 4.8	173.4 ± 13.6	4.6	0.443	175.6 ± 10.7	175.0 ± 10.3	−0.6	0.752
BMI (kg m^−2^)	22.9 ± 2.5	23.8 ± 2.5	0.9	0.497	25.3 ± 3.2	25.5 ± 3.4	0.2	0.737
Atrial fibrillation	0 (0)	0 (0)			9 (14.1)	8 (13.6)	−0.5	0.936
Hypertension	1 (16.7)	0 (0)	−16.7%	0.182	22 (34.4)	22 (37.3)	2.9	0.736
Diabetes mellitus type 2	0 (0)	0 (0)			8 (34.4)	7 (11.9)	−0.6	0.936
Dyslipidaemia	0 (0)	0 (0)			4 (6.3)	4 (6.8)	0.5	0.905
Coronary artery disease	0 (0)	0 (0)			1 (1.6)	1 (1.7)	0.1	0.954
TIA/CVA	0 (0)	1 (10)	10%	0.424	3 (4.7)	2 (3.4)	−1.3	0.716
COPD	0 (0)	0 (0)			0 (0)	0 (0)		
Smoking								
Currently	0 (0)	1 (10)	10%	0.424	10 (15.6)	9 (15.3)	−0.3	0.955
Previously	1 (16.7)	4 (40)	23.3%	0.330	30 (46.9)	25 (42.4)	−4.5	0.616
ASA class								
I	0 (0)	1 (10)	10%	0.424	7 (10.9)	6 (10.2)	−0.8	0.890
II	5 (83.3)	6 (60)	−23.3%	0.330	45 (70.3)	42 (71.2)	0.9	0.915
III	1 (16.7)	3 (30)	13.3	0.551	12 (18.8)	11 (18.6)	−0.1	0.998
Surgical discipline								
General	4 (66.7)	6 (60)	−6.7	0.790	54 (84.4)	51 (86.4)	2.1	0.746
Neurological	0 (0)	1 (10)	10%	0.424	5 (7.8)	3 (5.1)	−2.7	0.540
Gynaecological oncology	2 (33.3)	3 (30)	−3.3	0.889	2 (3.1)	1 (1.7)	−1.4	0.608
Vascular	0 (0)	0 (0)			1 (1.6)	2 (3.4)	1.8	0.512
Colon and rectal	0 (0)	0 (0)			1 (1.6)	1 (1.7)	0.1	0.954
Oral and maxillofacial	0 (0)	0 (0)			1 (1.6)	1 (1.7)	0.1	0.954
Surgical technique								
Open	2 (33.3)	7 (70)	36.7	0.152	39 (60.9)	34 (57.6)	−3.3	0.709
Laparoscopic	4 (66.7)	3 (30)	−36.7	0.152	25 (39.1)	25 (42.4)	3.3	0.709
Arterial line (left-sided)	4 (66.7)	8 (80)	13.3	0.551	50 (78.1)	45 (76.3)	−1.9	0.806
Finger cuff ipsilateral	4 (66.7)	10 (100)	33.3	0.051	53 (82.8)	46 (78.0)	−4.8	0.498

Data are presented as mean ± SD or *n* (%). The 70% cut-off was used to separate acceptable from poor HPI performance and evaluate possible differences in patient characteristics associated with these performances. The subsets of AUC values below 70% only include patients with at least one hypotensive event. In the invasive dataset, no hypotensive events were observed in 10 patients. In the noninvasive dataset, this absence was observed in 11 patients, with six patients overlapping between the two groups. AUC, area under the receiver-operating characteristics curve; BMI, body mass index; finger cuff, continuous noninvasive (radial arterial reconstructed) blood pressure; COPD, chronic obstructive pulmonary disease; CVA, cerebrovascular accident; arterial line, invasive (radial) arterial blood pressure catheter; HPI, Hypotension Prediction Index; TIA, transient ischemic attack.

In the invasive dataset, 70 patients experienced at least one hypotensive event, amounting to 389 events, with a median of 3 [1 to 8] events per patient and a cumulative time of 12.8 [4.3 to 42.3] min. These cumulative events had an area under the hypotension threshold (AUT) of 45.2 [10.7 to 143.6] mmHg × min and a time-weighted average (TWA) of 0.19 [0.05 to 0.60] mmHg. In the noninvasive dataset, 69 patients had at least one hypotensive event, resulting in a total of 412 events, a median of 3.5 [1 to 8] events per patient, with a cumulative time of 12.2 [3.8 to 37.2] min, an AUT of 61.9 [8.4 to 163.4] mmHg × min and a TWA of 0.29 [0.04 to 0.68] mmHg.

### Area under the receiver-operating characteristics curve comparisons

**Fig. 3:**
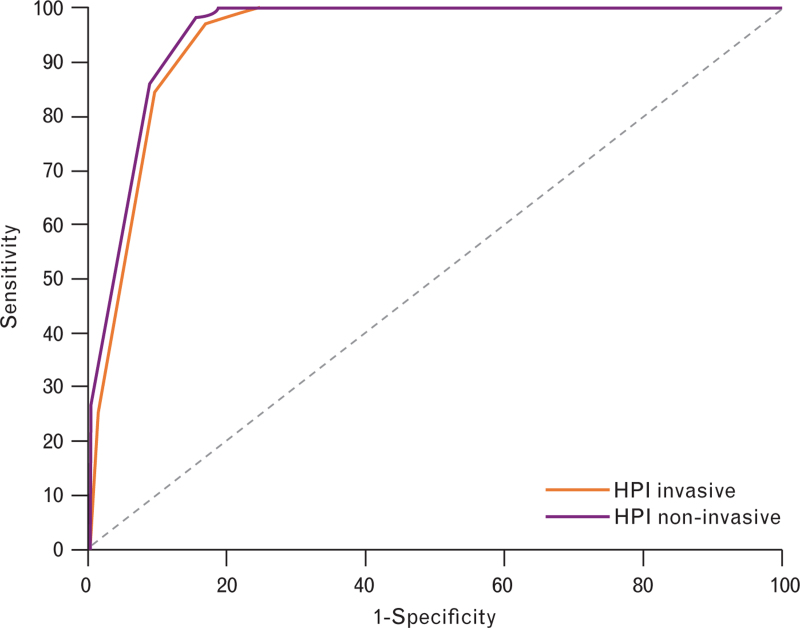
HPI compared between the invasive and non-invasive datasets.

The discriminatory ability of HPI, as measured by the AUC, showed no significant difference between the invasive and noninvasive datasets. The areas (95% CI) were 94.2 (90.5 to 96.8)% and 95.3 (90.4 to 98.2)%, with an estimated difference of 1.1 (−3.9 to 6.1)%; *P* = 0.673; Fig. 3. Tables 2 and 3 summarise the characteristics corresponding to the AUC values for the invasive and noninvasive datasets. These characteristics are provided for HPI thresholds ranging from 0 to 100, in increments of 5. Sensitivity and negative-predictive values (NPV) remain high across the entire range in both datasets, indicating that nearly all hypotensive events are preceded by an HPI alert above the respective threshold. Conversely, specificity and positive-predictive values (PPV) gradually increase at similar rates in both datasets.

Receiver operating characteristics curves are compiled by assigning true and false labels per HPI alerts and non-alerts evaluated against the occurrence or absence of a hypotensive event in the following 20-minute timeframe. HPI alerts were defined as exceeding the selected threshold for at least one minute until the value declined below that threshold for a minute. HPI, Hypotension Prediction Index; Invasive, invasive radial artery blood pressure; non-invasive, continuous non-invasive radial artery reconstructed blood pressure; min, minute(s).

**Table 2:** Area under the receiver-operating characteristics curve characteristics for the invasive dataset

HPI threshold	Sensitivity (%)	Specificity (%)	PPV (%)	NPV (%)
5	100 (100 to 100)	10.6 (7.3 to 14.2)	35.8 (29.9 to 42.5)	100 (100 to 100)
10	100 (100 to 100)	18.6 (14.8 to 22.5)	37.6 (31.0 to 44.0)	100 (100 to 100)
15	100 (100 to 100)	25.5 (21.8 to 29.6)	37.7 (31.4 to 44.3)	100 (100 to 100)
20	100 (100 to 100)	30.9 (26.9 to 35.1)	37.7 (31.4 to 44.6)	100 (100 to 100)
25	100 (100 to 100)	35.1 (30.9 to 39.5)	38.7 (32.3 to 45.2)	100 (100 to 100)
30	100 (100 to 100)	42.3 (37.6 to 47.1)	42.0 (35.1 to 48.2)	100 (100 to 100)
35	100 (100 to 100)	47.5 (43.2 to 52.0)	43.9 (37.4 to 49.9)	100 (100 to 100)
40	100 (100 to 100)	52.6 (48.1 to 57.3)	47.1 (40.6 to 53.7)	100 (100 to 100)
45	100 (100 to 100)	56.0 (51.7 to 60.3)	48.9 (42.4 to 55.6)	100 (100 to 100)
50	100 (100 to 100)	58.7 (54.4 to 62.8)	50.9 (43.5 to 57.7)	100 (100 to 100)
55	100 (100 to 100)	60.4 (56.1 to 64.6)	52.7 (45.7 to 59.8)	100 (100 to 100)
60	100 (100 to 100)	61.9 (58.0 to 66.5)	53.3 (46.1 to 60.1)	100 (100 to 100)
65	100 (100 to 100)	64.3 (60.2 to 68.7)	55.4 (48.6 to 62.9)	100 (100 to 100)
70	100 (100 to 100)	66.8 (62.8 to 71.2)	56.9 (49.4 to 64.1)	100 (100 to 100)
75	100 (100 to 100)	69.1 (65.2 to 73.2)	59.1 (51.7 to 66.0)	100 (100 to 100)
80	100 (100 to 100)	70.6 (66.5 to 74.7)	60.3 (52.7 to 67.5)	100 (100 to 100)
85	100 (100 to 100)	72.3 (68.5 to 76.3)	61.7 (54.6 to 68.4)	100 (100 to 100)
90	99.7 (99.1 to 100)	75.3 (71.7 to 79.2)	64.8 (58.1 to 71.3)	99.8 (99.5 to 100)
95	98.5 (97.0 to 99.5)	80.2 (76.3 to 83.6)	69.9 (63.9 to 75.7)	99.1 (98.3 to 99.7)

Data are presented as mean (95% CI). Each row represents the performance characteristics for a specific HPI threshold, where an alert is defined as exceeding that threshold for at least 1 min and continuing until the threshold is not exceeded for one minute. Alerts were classified as true positives if a hypotensive event was detected during the alert segment within 20 min. Similarly, false positives, true negatives and false negatives were assigned based on these criteria. Using these classifications, sensitivity [TP/(TP + FN)], specificity [TN/(TN + FP)], positive-predictive value (PPV) [TP/(TP + FP)] and negative-predictive value (NPV) [TN/(TN + FN)] were calculated. HPI, Hypotension Prediction Index; PPV, positive-predictive value; NPV, negative-predictive value.

**Table 3:** Area under the receiver-operating characteristics curve characteristics for the noninvasive dataset

HPI threshold	Sensitivity (%)	Specificity (%)	PPV (%)	NPV (%)
5	100 (100 to 100)	5.3 (2.7 to 8.3)	38.9 (31.5 to 47.5)	100 (100 to 100)
10	100 (100 to 100)	12.9 (8.4 to 18.0)	38.6 (30.7 to 46.8)	100 (100 to 100)
15	100 (100 to 100)	18.8 (13.4 to 24.1)	38.5 (30.9 to 46.4)	100 (100 to 100)
20	100 (100 to 100)	25.3 (19.3 to 30.9)	39.1 (31.7 to 47.3)	100 (100 to 100)
25	100 (100 to 100)	28.9 (23.0 to 34.5)	40.0 (31.9 to 48.3)	100 (100 to 100)
30	100 (100 to 100)	32.2 (26.1 to 37.6)	40.7 (32.4 to 49.2)	100 (100 to 100)
35	100 (100 to 100)	38.6 (33.2 to 43.2)	42.0 (33.9 to 50.4)	100 (100 to 100)
40	100 (100 to 100)	44.2 (39.4 to 48.8)	44.3 (36.4 to 52.4)	100 (100 to 100)
45	100 (100 to 100)	48.7 (43.8 to 53.0)	45.6 (37.4 to 54.2)	100 (100 to 100)
50	100 (100 to 100)	52.2 (47.4 to 56.5)	46.8 (38.7 to 55.9)	100 (100 to 100)
55	100 (100 to 100)	54.0 (49.5 to 58.5)	48.0 (39.4 to 56.6)	100 (100 to 100)
60	100 (100 to 100)	56.2 (51.8 to 60.4)	49.5 (40.9 to 58.3)	100 (100 to 100)
65	100 (100 to 100)	59.2 (54.5 to 63.5)	52.0 (43.6 to 60.4)	100 (100 to 100)
70	100 (100 to 100)	61.7 (56.6 to 66.3)	54.7 (46.0 to 62.8)	100 (100 to 100)
75	100 (100 to 100)	64.9 (60.1 to 69.6)	57.1 (48.8 to 64.9)	100 (100 to 100)
80	100 (100 to 100)	67.2 (62.2 to 72.3)	58.7 (50.9 to 66.9)	100 (100 to 100)
85	100 (100 to 100)	68.9 (63.8 to 73.8)	59.9 (51.6 to 68.6)	100 (100 to 100)
90	100 (100 to 100)	77.9 (74.2 to 81.7)	70.6 (63.9 to 76.7)	100 (100 to 100)
95	98.9 (97.8 to 99.7)	82.2 (77.9 to 86.1)	76.3 (69.9 to 82.0)	99.2 (98.3 to 99.8)

Data are presented as mean (95% CI). Each row represents the performance characteristics for a specific HPI threshold, where an alert is defined as exceeding that threshold for at least 1 min and continuing until the threshold is not exceeded for 1 min. Alerts were classified as true positives (TP) if a hypotensive event was detected during the alert segment within 20 min. Similarly, false positives (FP), true negatives (TN) and false negatives (FN) were assigned based on these criteria. Using these classifications, sensitivity [TP/(TP + FN)], specificity [TN/(TN + FP)], positive-predictive value (PPV) [TP/(TP + FP)] and negative-predictive value (NPV) [TN/(TN + FN)] were calculated. HPI, Hypotension Prediction Index; PPV, positive-predictive value; NPV, negative-predictive value.

### Time-to-event comparisons

The median time-to-event values ranged from approximately 2 to 7 min in both datasets. However, for higher HPI ranges, noninvasive HPI showed substantially longer time-to-event estimations, up to 60 s longer (Table 4).

**Table 4:** Time to event differences between the invasive and noninvasive datasets

HPI threshold	Time to event (min) Invasive	Time to event (min) Noninvasive	Time to event difference (min)	*P* value
5	7.00 (5.29 to 8.33)	6.17 (5.00 to 7.33)	−0.83 (−1.05 to −0.62)	<0.001
10	6.75 (5.50 to 8.17)	6.50 (5.42 to 7.33)	−0.25 (−0.44 to −0.06)	0.013
15	6.75 (5.50 to 7.58)	5.92 (4.96 to 7.00)	−0.83 (−1.00 to −0.66)	<0.001
20	6.67 (6.00 to 8.00)	6.33 (5.00 to 7.00)	−0.33 (−0.51 to −0.16)	<0.001
25	6.92 (5.75 to 7.67)	6.08 (4.83 to 6.83)	−0.83 (−1.01 to −0.66)	<0.001
30	6.67 (5.67 to 7.42)	5.92 (4.83 to 6.75)	−0.75 (−0.92 to −0.58)	<0.001
35	6.33 (5.33 to 8.00)	6.33 (4.67 to 7.33)	0.00 (−0.25 to 0.25)	1.000
40	5.17 (4.42 to 6.71)	6.33 (5.00 to 7.67)	1.17 (0.97 to 1.36)	<0.001
45	5.33 (4.33 to 6.21)	6.08 (4.92 to 7.00)	0.75 (0.58 to 0.92)	<0.001
50	5.17 (4.33 to 6.00)	5.75 (4.67 to 6.58)	0.58 (0.43 to 0.74)	<0.001
55	4.75 (4.00 to 5.67)	5.75 (4.50 to 6.58)	1.00 (0.83 to 1.17)	<0.001
60	4.75 (4.00 to 5.83)	5.00 (4.25 to 6.33)	0.25 (0.07 to 0.43)	0.007
65	4.67 (3.58 to 5.67)	4.67 (3.58 to 6.00)	0.00 (−0.17 to 0.17)	1.000
70	4.08 (3.33 to 5.08)	4.92 (3.67 to 5.67)	0.83 (0.66 to 1.01)	<0.001
75	3.83 (3.00 to 4.67)	4.58 (3.25 to 5.67)	0.75 (0.58 to 0.92)	<0.001
80	3.33 (2.67 to 4.00)	4.33 (3.33 to 5.08)	1.00 (0.86 to 1.14)	<0.001
85	3.25 (2.33 to 4.42)	4.17 (3.00 to 5.50)	0.92 (0.73 to 1.10)	<0.001
90	2.67 (2.00 to 3.33)	3.08 (2.00 to 3.83)	0.42 (0.28 to 0.56)	<0.001
95	2.00 (1.33 to 2.50)	2.33 (1.67 to 2.83)	0.33 (0.24 to 0.43)	<0.001

Data are presented as mean (95% CI). Time-to-event was calculated from alert onset to hypotension onset if the event occurred within 20 min, excluding alerts detected during hypotension or followed by a blood pressure-increasing intervention. HPI, Hypotension Prediction Index.

## Discussion

The AUC, reflecting discriminatory ability between events and nonevents, showed little discernible difference between invasively and noninvasively obtained HPI. Despite these similarities, the time from an HPI alert to hypotension onset was statistically longer in the noninvasive dataset, particularly for HPI thresholds above 70.

The similarity between invasive and noninvasive AUC values is consistent with the performance demonstrated in previous cohort studies evaluating noninvasive HPI in patients undergoing general surgery and gynaecological oncological surgery.^4,5^ Despite the suggested difference between time-to-event provisions within our datasets, both fell within reported ranges of time-to-event estimations in published validation studies.^4,16^ This demonstrates the added benefit of comparing HPI invasively and noninvasively in the same patient cohort, as it elucidates the differences that would otherwise go unnoticed. Although noninvasive blood pressure measurements show slightly lower values in some studies,^9^ the number of detected events varies between individuals in our cohort. Some have more events detected in the noninvasive dataset, whereas others have more in the invasive dataset. It is unclear which method more frequently misclassifies events. Despite these differences, the overall predictive capability for each HPI is similar, meaning that it detects hypotension within its own dataset effectively, but whether you are missing hypotension according to the other method will remain unknown in clinical practice.

The availability of differently obtained HPI values in identical patients also allowed us to look further than general differences. Examining patient characteristics for those with AUC values above or equal to 70%, below 70% and those without hypotension revealed that these subgroups contain, in some cases, over 50% different patients. In fact, the 70% threshold, generally considered the cut-off for acceptable AUCs, was surpassed in fewer patients when obtaining HPI noninvasively, as displayed in Table 1. This suggests that certain patient characteristics negatively affect noninvasive HPI without affecting invasive HPI and vice versa. However, none of the available characteristics seemed more prevalent in a particular group, preventing us from reporting associated factors. Also, no discernible differences in characteristics were found between the datasets when comparing patients with or without hypotension. Identifying these factors could aid in making informed decisions on whether to apply either invasive or noninvasive HPI.

An important limitation of the AUC arises when there are no events, as no true-positive or false-negative classes can be assigned. This automatically results in a sensitivity and AUC of 0%, rendering this method unsuitable for performance assessment in such situations.

Our study has limitations. Noninvasive HPI may provide significant benefits in settings where haemodynamic status can rapidly deteriorate and the initial need for an arterial line is not evident, such as in the postanaesthesia care unit, emergency department or cardiac care unit. Notably, these patients are typically not sedated, making direct extrapolation of our study findings to these settings not entirely suitable. Although there are intriguing indications of HPI's predictive ability in awake patients undergoing caesarean delivery under spinal anaesthesia,^3^ it would have been advantageous to include nonsedated patients for a broader validation. Secondly, in the absence of annotated data regarding specific treatments, we employed a filtering process to identify rapid changes in MAP indicative of haemodynamic intervention. However, it is important to note that this approach may have resulted in missed interventions. Thirdly, it is not evident whether the invasive or noninvasive blood pressure dataset should be considered the gold standard when determining hypotensive events. Both datasets often recognise hypotension simultaneously, but it is unclear which is correct when only one does. We considered using invasive blood pressure as the gold standard, comparing HPI from both datasets against it. However, as HPI depends on the blood pressure data it receives, noninvasive HPI would be inherently disadvantaged. If invasive blood pressure did not detect hypotension while noninvasive did, noninvasive HPI would be wrongly classified as false-positive. To avoid such discrepancies, we decided to evaluate HPI's predictive capability against its corresponding blood pressure, aligning with clinical practice. Lastly, our data collection did not include secondary variables that are displayed concurrently with HPI. This could have provided valuable insights into whether the underlying causes of hypotension as portrayed by these variables were similar.

## Conclusion

In conclusion, clinicians can reliably access noninvasive HPI during noncardiac surgery, and not only when an arterial line is necessary. The accuracy of HPI probabilities is generally comparable between the two options and may even allow for additional response time when noninvasively obtained.
